# Influence of surface wettability on electrostatic fog collection efficiency using polytetrafluoroethylene-coated carbon papers

**DOI:** 10.1098/rsos.250914

**Published:** 2025-11-05

**Authors:** Qiongdan Xie, Fei Zheng, BeiEr Yang, YongHao Han

**Affiliations:** ^1^ School of New Energy and Intelligent Connected Vehicle, Sanya University, Sanya, Hainan 572000, People’s Republic of China; ^2^ School of International Hospitality Management, Sanya University, Sanya, Hainan 572000, People’s Republic of China

**Keywords:** fog collection, superhydrophobic, carbon paper, electrostatics

## Abstract

Electrostatic fog collection offers a low energy cost and highly efficient method for fog collection; however, the correlation between efficiency and collector surface wettability has not yet been fully explored. This study examines the effectiveness of polytetrafluoroethylene (PTFE)-coated carbon papers in electrostatic fog collection and reveals that their performance is comparable to that of hydrophilic carbon papers. These findings suggest that PTFE coating on the carbon paper surface has a minimal influence on the electrostatic fog collection efficiency. On hydrophilic carbon paper, deposited fog droplets coalesce into a continuous water film that subsequently drains, whereas on hydrophobic carbon paper, the droplets aggregate into discrete spherical forms. This variation in wetting behaviour exerts nearly no influence on the overall fog collection efficiency. Furthermore, the efficiency of electrostatic fog collection is unaffected by charge polarity, with both negative and positive high voltages yielding comparable fog collection efficiencies. Collection efficiency is directly correlated with electrostatic current and shows an exponential relationship: collection efficiency rises sharply with increasing current before reaching a near-plateau. In addition, a thermal camera was utilized to map the zone where the fog stream is collected on the carbon paper, and it was shown that a strong electric field could cause segregation of the charged fog stream.

## 1. Introduction

Sustainable Development Goal Six (access to water and sanitation for all) by 2030 still faces unprecedented challenges owing to the impact of global climate change. The challenges are even more daunting for countries or regions that lack traditional freshwater resources (e.g. rivers, lakes, groundwater and rainwater). These resources are vital for meeting various human and ecological needs, including drinking water, irrigation and industrial applications, to maintain biodiversity. Fog, which consists of fine water droplets suspended in the atmosphere, is another freshwater resource. Harnessing fog water provides an alternative route for supplying fresh water to water-scarce communities and regions [1]. Fog harvesting technology can be divided into two main types: passive and active fog harvesting [2,3]. Passive fog harvesting relies on the collection of fog particles through inertial collision, a process in which tiny water droplets collide with a collecting surface and coalesce into larger droplets [4]. Active fog harvesting systems generally demand additional energy, either to capture fog droplets or to release the collected water. Various approaches have been studied, including active adsorption [5], the use of temperature differentials [2] and electrostatic fog collection [6]. Of these approaches, electrostatic collection employs ultrahigh-voltage fields to ionize air at the emitter, imparting charge to fog droplets. The charged droplets drift towards a grounded conductor and coalesce, with electrostatic forces overcoming aerodynamic drag to markedly improve collection efficiency [7].

In general, the fog collection process consists of two key stages: droplet capture and surface-mediated liquid transport. While the capture mechanisms of both passive and electrostatic systems are well established [7,8], the subsequent transport is governed largely by surface wettability, which dictates droplet adhesion, coalescence and drainage. The role of wettability in determining fog collection efficiency remains under active debate. In passive systems, extensive studies have shown that tailoring surface properties can significantly improve drainage. For instance, Azeen *et al.* demonstrated that hydrophobic materials outperform hydrophilic ones when applied to filament and harp structures as the fog collectors [9–11]. Similarly, coating polyolefin Raschel mesh with a hydrophobic fluorodecyl polyhedral oligomeric silsesquioxane/poly(ethyl methacrylate) layer yielded a fivefold increase in efficiency by reducing contact angle hysteresis (CAH) and preventing clogging [12]. However, under high-wind conditions, superhydrophobic surfaces may lead to droplet re-entrainment, reducing the overall water collection efficiency [13]. Conversely, hydrophilic surfaces can improve droplet nucleation and coalescence, thereby enhancing the initial capture efficiency. Nevertheless, they are prone to clogging, which can obstruct airflow and diminish the overall performance [14]. These findings underscore the importance of optimizing the surface wettability to balance water drainage with minimal interference in fog capture. Current research on passive fog collection is increasingly directed towards the development of (super)hydrophilic–(super)hydrophobic hybrid [15] or Janus multifunctional collection surfaces [16,17]. By integrating contrasting wettability regions, these surfaces aim to overcome the intrinsic trade-offs of single-wettability designs, thereby improving overall collection efficiency.

The efficiency of electrostatic fog collection systems is influenced by several factors, including the configuration and geometry of the collector [18–22]. The effects of the applied voltage, wind direction, velocity of the airflow, concentration of fog and temperature have been previously reported [7,23,24]. However, previous studies have primarily focused on collector geometry and structural design, whereas the influence of surface wettability on collection performance has received comparatively little attention. In this work, electrically conductive carbon paper was employed as a model collector, with its surface wettability adjusted either by applying polytetrafluoroethylene (PTFE) coatings or by plasma treatment. The modified substrates exhibited contact angles ranging from approximately 15° to 150°, thereby spanning both hydrophilic and superhydrophobic regimes. In addition, the hydrophobic carbon paper surfaces exhibited pronounced CAH. Portions of this study were previously presented at the 2024 International Symposium on Green and Sustainable Technology [25]. The present paper provides an expanded dataset and a more comprehensive analysis of electrostatic fog collection. We investigated the influence of surface wettability on collection efficiency, as well as the effect of high-voltage electrostatic fields, with particular attention to the comparative roles of positive and negative polarities. In addition, the impact of collection distance was examined, as variations in field strength and uniformity were found to alter droplet trajectories and, consequently, overall efficiency. The findings of this study provide insights for optimizing electrostatic fog collection systems—particularly the charge-receiving surfaces—and may contribute to the development of more practical, lightweight and efficient electrostatic collection designs.

2. Materials, instruments and methods

### 2.1. Materials and instruments

Carbon papers coated with different concentrations of PTFE (ranging from 5 to 40 wt%) were procured from Suzhou Sinero Technology Co. Ltd for experimental use. The 0 wt% PTFE carbon paper was treated with sub-atmospheric pressure glow discharge plasma (Nanjing Suman Plasma Technology Co. Ltd) to enhance its surface hydrophilicity. The treatment was performed in air at a working pressure of 50 Pa for 2 min and repeated 15 times. A PTFE film with a thickness of 2 mm was purchased from Chengguang Plastic (China) and used as received. The high-voltage power supplies were obtained from Dongwen High Voltage Power Supply (Tianjin, China), including two models: a negative high-voltage unit capable of delivering up to –50 kV at a maximum current of 1 mA and a positive high-voltage unit with a maximum output of +50 kV and 1 mA current limit. The static water contact, advancing and receding angles of the PTFE-coated carbon papers were measured using a modified contact angle goniometer (Chengde Tuoei Technology Co. Ltd, Hebei, China). The morphology of the water droplets collected on the surfaces was examined using a P-60V optical microscope and USB industrial high-definition CCD camera (Kunshan Gaopin Precision Instrument Co. Ltd, Jiangsu, China). The electrical resistivity of the carbon paper was characterized using a four-point probe system (Model TRS-8, Guangzhou 4Probs Co. Ltd, China). The PTFE content in hydrophobic carbon paper was quantified by thermogravimetric analysis (TGA; Mettler Toledo TGA2). Approximately 4 mg of sample was placed in an alumina pan and heated from 30 to 700 °C at 10 °C min⁻¹ under nitrogen protection (approx. 50 ml min⁻¹). Mass loss and derivative thermogravimetry curves were recorded to determine decomposition behaviour and final residue. Additionally, a handheld thermal camera (Model H21pro, HIKMICRO, China) was used to visualize the deposition zone of the charged fog stream on the carbon paper surface. A K-type thermocouple (Model TA612C, TASI, China) was used to measure the carbon paper surface temperature and cross-check thermal camera readings. A high-speed camera (YVSION OSG030-815UMTZ, Machine Vision Company, China) recorded fog trajectories in a fog collection process with a frame rate of 3000 f.p.s. X-ray photoelectron spectroscopy (XPS) experiments were carried out with an X-ray photoelectron spectrometer (Thermo Fisher Scientific, ESCALABXi+, USA) using 200 W Al Kα radiation. The inelastic background of C 1s was removed using Shirley’s method. All binding energies were corrected by calibration on the graphite C 1s peak at 284.80 eV. Attenuated total reflectance Fourier transform infrared (ATR-FTIR) spectroscopy of the carbon papers was performed using an FTIR spectrometer (FOLI10-R-T, INSA Optics, China) with a wavenumber range of 4000–500 cm⁻¹, a resolution of 4 cm⁻¹ and 32 averaged scans.

### 2.2. Methods

**Figure 1.**
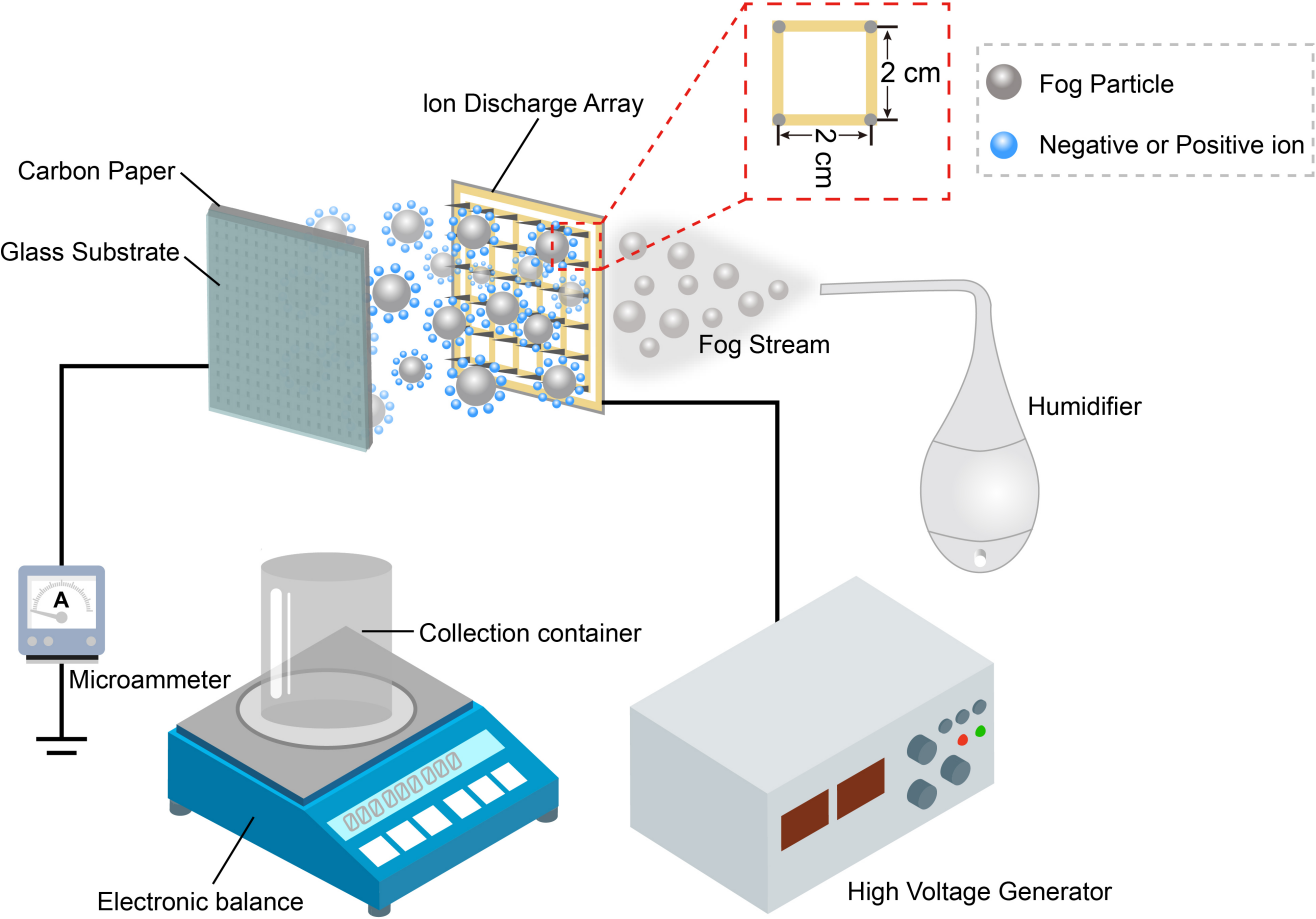
Schematic illustration of the electrostatic fog collection system. A photograph of the experimental set-up is presented in electronic supplementary material, figure S1.

Ultrapure water was used to prevent interference from other factors in natural fog (e.g. contamination) [26]. The distances between the carbon paper and electrostatic discharge copper needle tips were 2, 3 and 4 cm, respectively. The applied voltage ranged from ±3 to ±13 kV. A schematic representation of the experimental design is shown in figure 1, and a photograph of the actual set-up is shown in electronic supplementary material, figure S1. In this set-up, a humidifier was used to produce a continuous fog stream. Three fog streams of different flux speeds were applied, which were 7.9 ± 0.1, 2.8 ± 0.1 and 1.2 ± 0.1 g min^−1^. The copper needles were oriented perpendicular to the collection surface. The fog stream passes through the copper needle array, and the aerosolized droplets emitted from the humidifier hose effectively enter the electric field, allowing for an accurate evaluation of the overall collection efficiency.

Carbon paper (i.e. fog collector) was cut to 10 × 10 cm^2^, supported with a 10 × 10 cm^2^ glass plate and electrically grounded. A microammeter (Model S02, ZhongXinDa Electrics Co., China) was connected in series between the carbon paper and the ground wire to measure the electrostatic current received by the carbon paper. The charged fog stream was directed onto the carbon paper surface, where it was collected. The resultant water was collected in a clean flask and quantified using an analytical balance. As shown in the inset image in electronic supplementary material, figure S1, the electrode consisted of 36 sharp copper needles uniformly distributed across a 10 × 10 cm^2^ perforated circuit board, with a spacing of 2 cm between adjacent needles. For every droplet deposition condition, the deposition process continued for the first 5 min to reach a stable deposition state, after which the collected water was collected continuously at intervals of 3 min to obtain the collection efficiency (grams per minute). Each collection experiment was repeated three times to obtain the average collected water mass and corresponding s.d.

## 3. Results and discussion

### 3.1. Characterization of carbon papers

**Figure 2.**
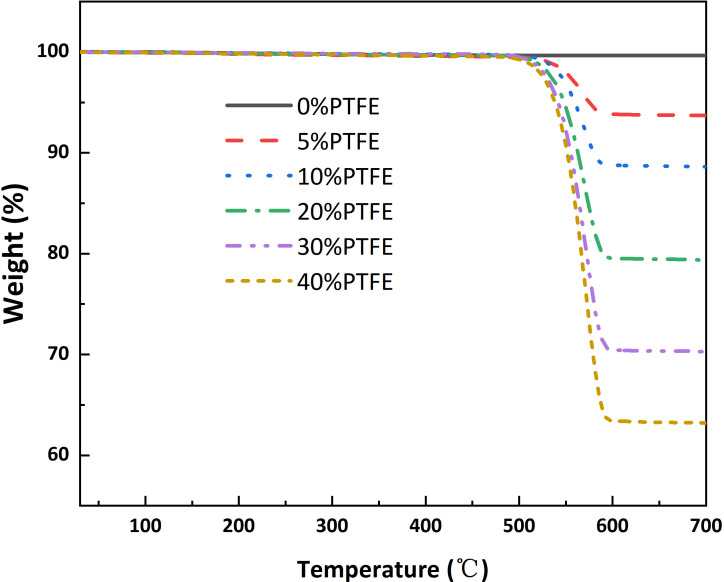
TGA results of the carbon papers.

The PTFE content in the carbon papers was quantified by TGA. As shown in figure 2, the carbon paper matrix remains stable across the entire test temperature range (30–700 °C), whereas the PTFE component exhibits a distinct degradation step starting at approximately 500 °C. Based on the corresponding weight loss, the measured PTFE contents were 6.3, 11.4, 20.6, 29.7 and 36.8% for the nominal 5, 10, 20, 30 and 40% PTFE samples, respectively.

**Figure 3.**
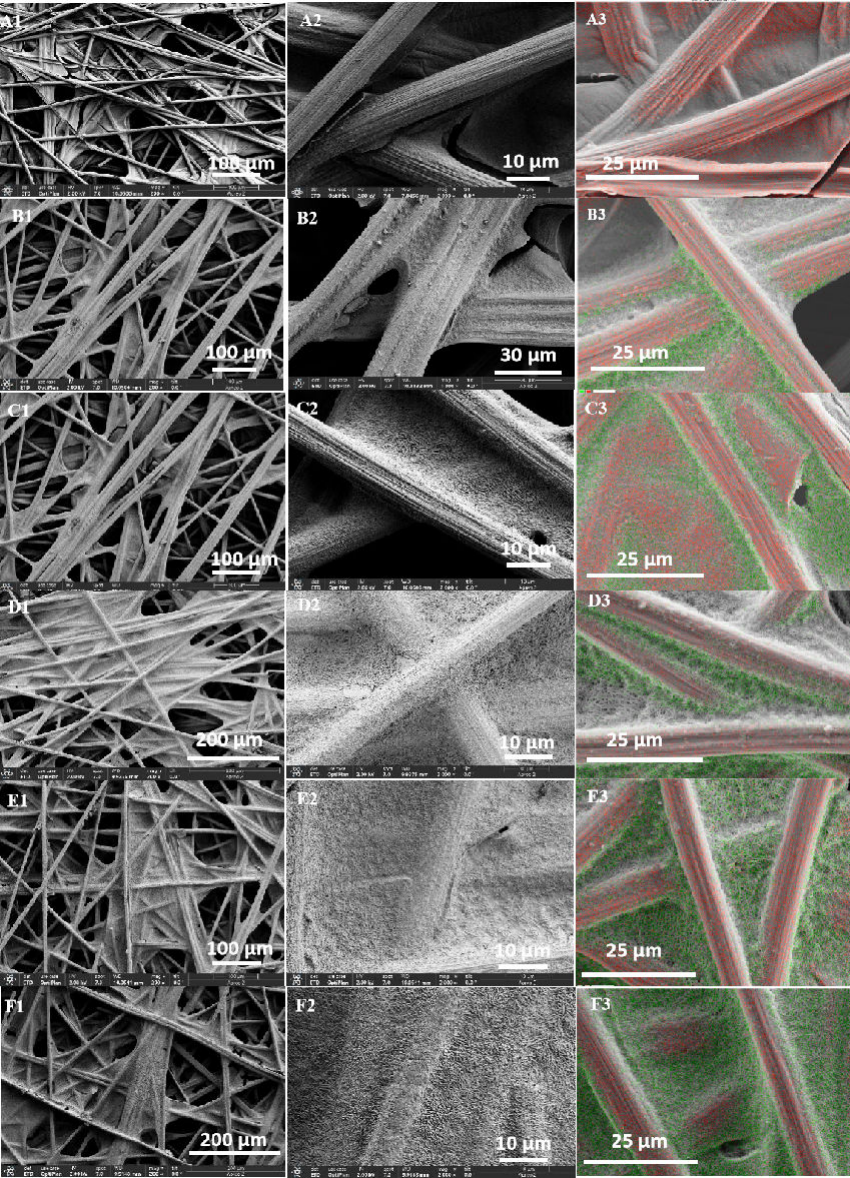
SEM images and EDS elemental mapping of carbon paper with varying PTFE content. (A) Untreated, (B) 5% PTFE, (C) 10% PTFE, (D) 20% PTFE, (E) 30% PTFE, (F) 40% PTFE. Columns: left—SEM morphology; middle—SEM higher magnification; right—EDS elemental maps (C: red, F: green).

The surface microstructure and morphology of the carbon papers were examined using scanning electron microscopy (SEM). To assess the distribution of the PTFE coating, energy-dispersive spectroscopy (EDS) mapping was performed, where carbon and fluorine are represented in red and green, respectively (figure 3, third column). Overall, figure 3 provides a comprehensive overview of the surface morphology and elemental composition of the different samples. Specifically, figure 3A displays the microstructure of the untreated carbon paper. In figure 3A1, the pristine carbon paper exhibits a highly porous structure composed of intersecting carbon fibres. Local regions of the fibres are covered with thin carbon film patches. As shown in figure 3A2, the fibres have diameters of approximately 7–10 µm, and their surfaces display fine longitudinal grooves aligned along the fibre axis. In the EDS mapping of the pristine carbon paper (figure 3A3), only the red signal corresponding to carbon was detected. The SEM images in figure 3B1,C1,D1,E1,F1 show that the porous architecture of the carbon papers was largely preserved, with the PTFE coatings not fully occluding the pores. PTFE deposits were observed on both the carbon fibres and the patch-like carbon film regions, but their distribution was distinctly uneven. As the PTFE content increased, the coating in the patch regions became noticeably thicker and developed increasingly diverse surface textures, whereas the coatings on the fibre surfaces remained relatively thin. This non-uniform distribution likely arises from the PTFE deposition process during fabrication.

The EDS results for the PTFE-coated carbon papers (figure 3B3,C3,D3,E3,F3) provide further insight into the distribution of PTFE. With increasing PTFE content, the intensity of the green signal in the EDS maps correspondingly increased. Notably, the fibre surfaces exhibited much lower PTFE concentrations compared with the carbon patch regions and the junction zones between fibres and patch films. Fluorine was almost undetectable on the curved outer layers of the fibres, suggesting that PTFE preferentially accumulated beneath the fibre surface layers. Elemental weight ratios were quantified from the EDS spectra after subtracting the gold contribution introduced during SEM sample preparation. The corrected fluorine weight percentages were determined to be 26.8, 36.6, 37.2, 44.2 and 52.0% for the carbon paper composites containing 5, 10, 20, 30 and 40 wt% PTFE, respectively (electronic supplementary material, table S1). It should be noted, however, that owing to the intrinsic porosity of the carbon paper and the non-uniform distribution of PTFE within the matrix, the fluorine contents obtained from EDS deviate from the actual bulk PTFE fractions.

**Figure 4.**
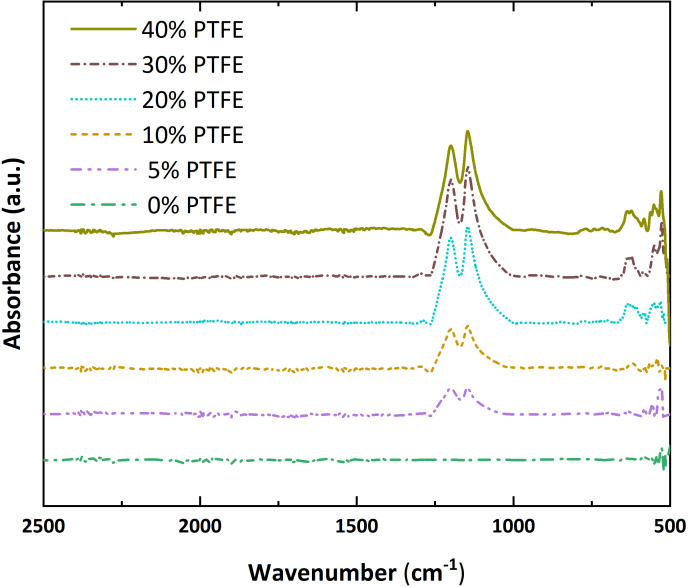
ATR-FTIR spectra (shifted for clarity) of the carbon papers.

To further investigate the surface chemical composition and confirm the presence of PTFE in the carbon paper composites, ATR-FTIR analysis was conducted. In figure 4, ATR-FTIR spectra of the carbon paper composites containing 0–40 wt% PTFE show the progressive appearance of PTFE characteristic bands. The doublet at ≈1200 and ≈1150 cm⁻¹ is assigned to asymmetric and symmetric C–F stretching of –CF₂ and is absent in the spectrum of the PTFE-free sample. Additional CF₂ bending and skeletal modes at ≈650–620 cm⁻¹ and ≈520–480 cm⁻¹ increase in intensity with PTFE content [27,28]. No new absorption bands or significant peak shifts were observed, suggesting that PTFE is physically mixed into the matrix without forming new covalent bonds under the applied processing conditions.

**Figure 5.**
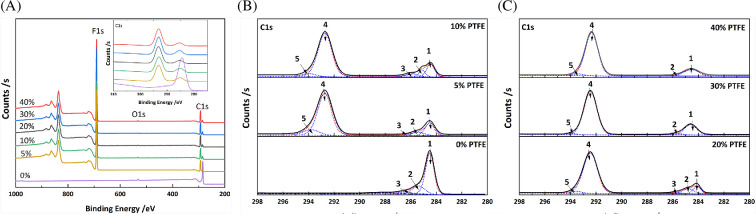
(A) XPS survey spectra (offset for clarity) showing C 1s, O 1s and F 1s contributions; inset: comparison of the C 1s spectra for PTFE-free and PTFE-coated carbon papers. (B) Deconvolution of the C 1s spectra for 0, 5 and 10 wt% PTFE carbon papers. (C) Deconvolution of the C 1s spectra for 20, 30 and 40 wt% PTFE carbon papers.

XPS analysis was carried out to further elucidate the surface composition of carbon papers (figure 5). The C 1s peak at 284.8 eV was used as the internal reference for spectral calibration. In the survey spectra, the PTFE-free carbon paper (0 wt%) exhibits only the C 1s signal at approximately 284.8–260.0 eV, characteristic of graphitic carbon, accompanied by a weak O 1s peak at approximately 533 eV, which can be ascribed to surface-adsorbed oxygen species or native oxidation. Notably, the weak O 1s peak remains in the spectra of PTFE-coated carbon papers, suggesting that surface-adsorbed oxygen species or native oxidation remains after PTFE coating. The PTFE-coated samples show a distinct F 1s signal (approx. 689 eV) and a splitting of the C 1s peak into multiple components (inset of figure 5A), reflecting the incorporation of the PTFE layer onto the carbon paper surface. For the PTFE-free sample, the C 1s region is dominated by a single peak at approximately 284.8–286.0 eV, corresponding to C–C/C=C in graphitic carbon. With increasing PTFE content, new C 1s contributions appear at higher binding energies (approx. 289–292 eV), which are attributed to fluorinated carbons (CF₂ and CF₃ groups). Deconvolution of the C 1s spectra (figure 5B,C) reveals two main groups of peaks: the first group consists of graphitic carbon at approximately 284.8 eV (peak 1) [29], defect-related carbon (amorphous carbon or disorder carbon) at approximately 285.7 eV (peak 2) [30] and carbon bonded to hydroxyl groups (C–OH) at approximately 286.2 eV [31]. The second group corresponds to higher binding energy peaks arising from fluorinated carbons introduced by PTFE. Specifically, peak 4 (approx. 289.0–290.0 eV) is assigned to –CF₂ groups, while peak 5 (approx. 291.5–292.0 eV) is attributed to –CF₃ groups [29]. In contrast, the C–O component (peak 3) decreases with increasing PTFE content and becomes negligible in the 30 and 40 wt% PTFE composites.

The surface chemical compositions of the carbon papers, as determined by XPS, are summarized in table 1. Regardless of PTFE loading, the elemental distributions of the PTFE-modified samples remain comparable, with F 1s accounting for 64.3–66.2% and C 1s for 33.0–34.9%. The O 1s contribution decreases from 2.0% in the PTFE-free sample to less than 1% in the PTFE-loaded carbon papers, suggesting the removal of oxygen species or native oxidation products during the process of PTFE deposition. The F/C atomic ratio of the PTFE-modified carbon papers falls within the range of 1.8–2.0. These results indicate an uneven distribution of the PTFE coating on the carbon paper surface, with PTFE predominantly concentrated at the top of the substrate. The F/C atomic ratio reflects contributions from both the C 1s signal of PTFE and that of the exposed carbon paper substrate. To better understand the distribution of PTFE on the top surface layer, deconvolution results of the C 1s spectra were employed to separately quantify the contributions from PTFE and the carbon paper substrate. The resulting C 1s_PTFE_/C 1s_substrate_ ratio ranges from 3.6 to 5.5. These atomic ratios are consistent with the SEM–EDS observations in figure 3, which show that the graphite carbon paper fibres remain largely exposed on the top layer.

**Table 1.** The XPS element composition of carbon papers.

PTFE (%)	C 1s (%)	F 1s (%)	O 1s (%)	F 1s/C 1s	C 1s_PTFE_/C 1s_substrate_
0	98.0	—	2.0	—	—
5	34.1	64.9	0.0	1.9	4.1
10	34.9	64.3	0.8	1.8	3.6
20	33.0	66.4	0.6	2.0	4.9
30	33.8	65.4	0.8	1.9	4.3
40	33.1	66.2	0.7	2.0	5.5

Notably, the surface compositions obtained by EDS and XPS differ. To correctly interpret the complementary information provided by the two techniques, it is essential to recognize their fundamental differences. EDS probes a relatively large interaction volume, with a typical sampling depth of approximately 0.1–3 µm, making it suitable for bulk compositional analysis [32]. In contrast, XPS is highly surface-sensitive, analysing only the top approximately 5 nm of a material, and is therefore well suited for investigating surface chemical states [33]. Consequently, EDS primarily reflects bulk composition, whereas XPS provides surface-specific chemical information from the outermost layer.

To study the influence of the PTFE coating on the conductivity of carbon paper, the volume resistivity of the carbon papers was measured using a four-point probe resistivity measurement instrument, and the results are summarized in electronic supplementary material, table S2. Relative to pristine carbon paper, all hydrophobic carbon papers exhibited comparable through-plane resistivity values of approximately 80 mΩ cm, regardless of PTFE content. This finding suggests that the PTFE coating has a negligible effect on the resistivity of the carbon paper. This finding is consistent with the surface microstructure and PTFE distribution on the modified carbon paper, as shown in figure 3. The PTFE coating on the top-layer carbon fibres was very thin, leaving the arc surfaces of the carbon fibres exposed. Consequently, the hydrophobic coating had a minimal impact on the resistivity of the carbon paper. It is worth noting that the four-point probe resistivity measurement, which employs four tiny needles that make tight contact with the sample surface, may pierce through the PTFE coating layer, resulting in similar resistivity values.

### 3.2. Surface wettability characterization

**Figure 6.**
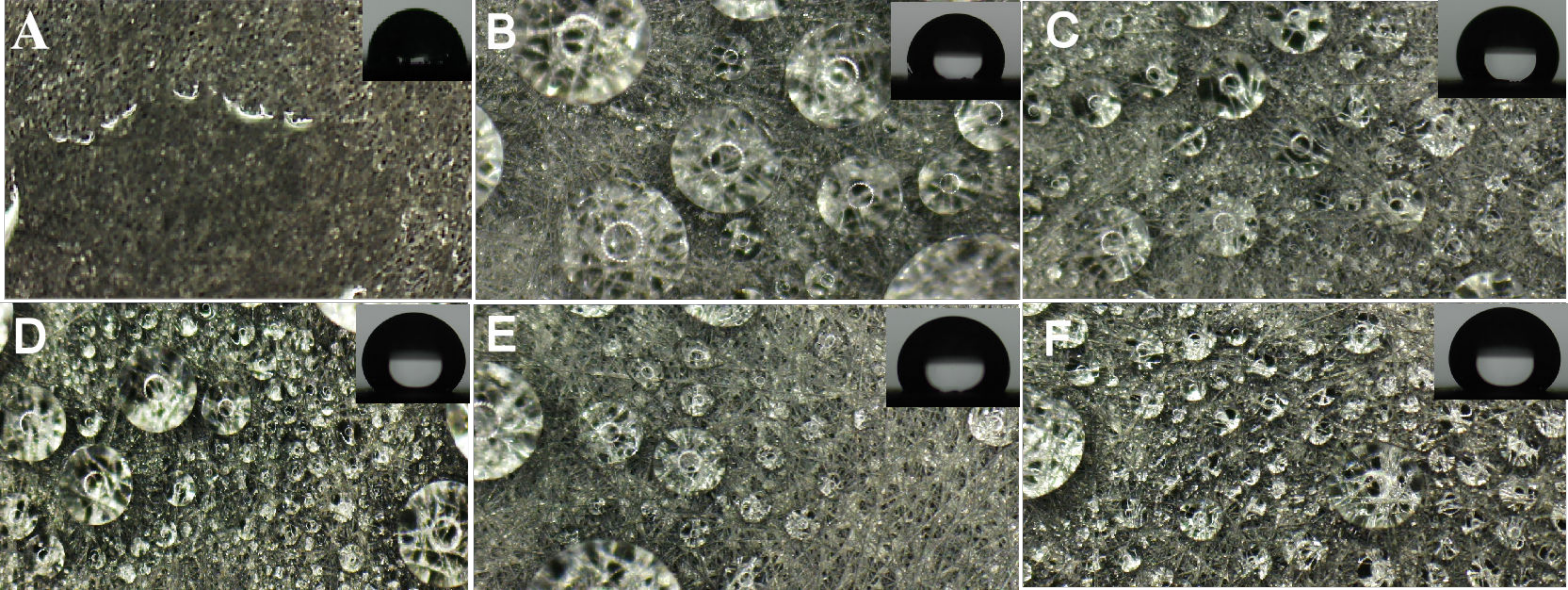
Optical microscopic images (×21) of deposited water drops on carbon paper surfaces. The insets depict the contact angle, measured with a 5 μl ultrapure water drop. (A–F) Carbon papers of 0, 5, 10, 20, 30 and 40% PTFE, respectively.

Figure 6 presents optical micrographs of water droplets on the carbon paper surfaces, with inset images (upper right) showing the static contact angle of a 5 µl droplet for each sample. Despite the intrinsic hydrophobicity of PTFE-coated carbon papers, fog droplets are deposited on their surfaces under electrostatic conditions. As shown in figure 6A, the PTFE-free sample exhibits a low contact angle (approx. 70°), with the collected water spreading into a continuous layer. This behaviour indicates that the porous structure of the uncoated carbon paper promotes water infiltration into the material interior. In contrast, the inset images in figure 6B–F display markedly higher static contact angles of 105 ± 5°, 140 ± 5°, 145 ± 5°, 148 ± 3° and 150 ± 5° for carbon papers coated with 5, 10, 20, 30 and 40 wt% PTFE, respectively. Notably, surfaces with contact angles exceeding 150° are generally classified as superhydrophobic [34]. These results indicate that the PTFE coating progressively enhanced the surface static hydrophobicity, with higher contact angles emerging at higher PTFE coating percentage.

On PTFE-coated carbon paper, collected fog droplets form near-spherical shapes with diameters ranging from sub-millimetre to millimetre scales. The combination of surface hydrophobicity and underlying porosity restricts droplet spreading and coalescence, maintains high contact angles and promotes droplet retention in a discrete form.

In addition to the static contact angles, the advancing and receding contact angles of the carbon paper are summarized in table 2. For the pristine carbon paper, the advancing contact angle ranged from 105° to 110°, notably higher than the static contact angle of approximately 70°. This difference arises because the advancing contact angle accounts for the surface roughness, chemical heterogeneity and dynamic nature of liquid spreading [35]. The receding contact angle was measured by withdrawing water from the droplet via a needle connected to a peristaltic pump, recording the angle as the liquid–solid contact line receded. However, for the pristine carbon paper, the droplet did not de-wet; instead, the contact angle continuously decreased until droplet detachment occurred, preventing the determination of the receding angle. This non-dewetting behaviour is attributed to the capillary effect induced by the high porosity of carbon paper [36]. For PTFE-modified carbon papers, the advancing contact angles (*θ*_a_) were 150–160°, while the receding angles (*θ*_r_) were much lower at 55–85°. Static hydrophobicity is characterized by the static contact angle, whereas dynamic hydrophobicity is evaluated using the rolling/sliding angle (*α*) and CAH. *α* denotes the minimum tilt at which a droplet detaches from the surface, and CAH reflects the extent of droplet pinning. As shown in table 2, all samples displayed significant CAH, indicative of strong liquid–solid interfacial pinning. To elucidate the significant contact hysteresis effect, electronic supplementary material, figure S2, shows that a 5 µl water droplet can remain adhered to the vertically oriented carbon paper surfaces. The underlying cause of this pinning effect is attributed to the heterogeneous surface components, as shown in figure 3. Specifically, the modified carbon paper surface incorporates heterogeneous elements that induce a ‘gecko’-like wetting state [37], in which water droplets exhibit selective adhesion to hydrophilic carbon fibres. This configuration promotes droplet attachment, while the porous architecture and hydrophobic PTFE domains suppress lateral spreading. Consequently, the surface displays a high static contact angle together with pronounced CAH.

**Table 2.** Static contact angles, advancing angles and receding angles of the carbon papers.

surface wettability	0% PTFE	5% PTFE	10% PTFE	20% PTFE	30% PTFE	40% PTFE
contact angle (CA) (°)	~70	105 ± 5	140 ± 5	145 ± 3	148 ± 3	150 ± 5
advancing angle (*θ*_a_) (°)	105–110	150–160	155–160	152–155	150–155	150–158
receding angle (*θ*_r_) (°)	—	70–85	50–60	55–80	55–70	50–80
CA hysteresis (*θ*_a_ − *θ*_r_) (°)	—	65–90	95–110	72–100	80–100	70–108

During fog collection, as additional water accumulates, the droplet eventually reaches a critical mass that overcomes gravitational forces, causing it to detach and roll off the vertically positioned carbon paper surface. Furmidge [38] proposed a relationship linking the rolling angle/sliding angle, drop mass to CAH, expressed as


(3.1) $$mg\sin\left(\alpha \right)=\omega \gamma (\cos\theta_{a}-\cos\theta_{r}),$$

where *m* is the droplet mass, *g* is gravitational acceleration, *γ* is the liquid–vapour surface tension and *ω* is the droplet width in the direction of sliding. This equation provides a quantitative framework for describing droplet mobility on solid surfaces in terms of surface wettability. According to table 2, the CAH values are comparable across all PTFE-modified carbon papers. Consequently, as indicated by equation (3.1), the critical mass of a water droplet required to detach from the vertically oriented carbon paper is expected to be similar among the different samples. For the unmodified carbon paper, water droplets do not de-wet from the surface; instead, the deposited droplets coalesce into a continuous water film, which eventually drips downward. The similar CAH values observed for all PTFE-modified carbon papers can be explained by XPS results, which show comparable PTFE enrichment in the outermost surface.

### 3.3. High-speed camera characterization

Based on the surface analysis of the carbon papers, the exposed carbon fibre surfaces serve as charge-receiving sites during electrostatic fog collection. As shown in figure 1, when fog particles enter the electric field near the discharging electrode needles, they acquire charges and are driven towards the surface, where they discharge, deposit and collide with previously collected droplets. This process leads to the formation of larger droplets or the development of a continuous water film. Once the deposited water reaches a critical mass or droplet size, it detaches and drips downward. To directly visualize the fog stream behaviour under different electric field conditions, a high-speed camera was employed to capture the trajectories of the fog stream with and without an applied voltage (0–12 kV) at a collection distance of 2 cm.

**Figure 7.**
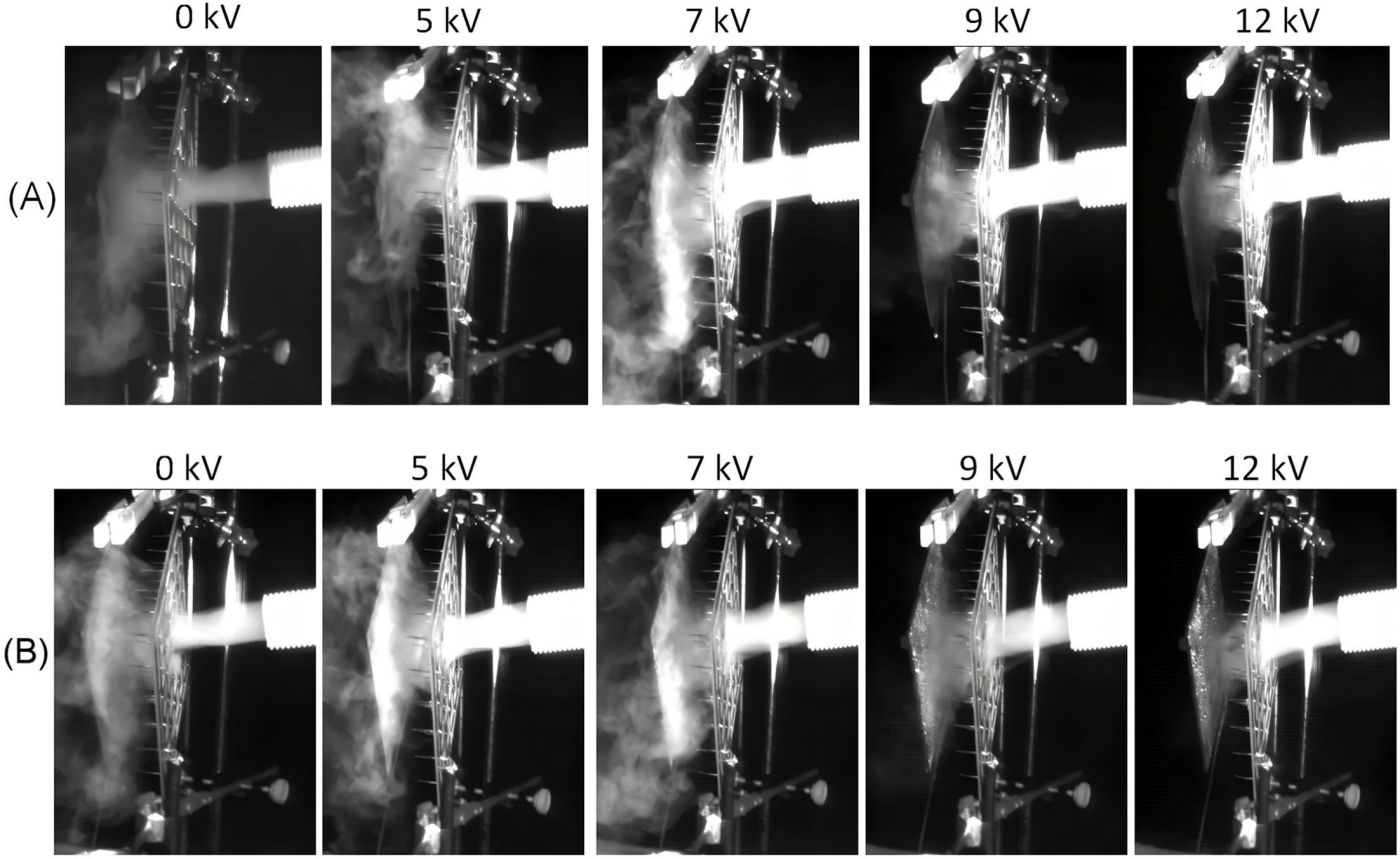
High-speed camera snapshots illustrating fog stream directed towards carbon papers subjected to different applied voltages: (A) hydrophilic carbon paper (0% PTFE) and (B) hydrophobic carbon paper (40% PTFE). Corresponding recordings for additional PTFE-coated surfaces are available in the electronic supplementary material.

Representative snapshots of the fog stream under different voltages are shown in figure 7. At relatively low voltages (0, 5 and 7 kV), the fog stream was only weakly influenced by the applied field and readily bypassed the collection surfaces, regardless of whether the carbon paper was hydrophilic or hydrophobic. This deflection away from the surface led to poor interception of fog droplets and, consequently, low collection efficiency. As the voltage was increased to 9 and 12 kV, a distinct change in trajectory was observed. The fog stream became confined between the needle–plate electrode and the carbon paper, significantly enhancing the droplet capture. At 12 kV, the confinement effect was further strengthened, with the stream increasingly directed towards the central region of the carbon paper. The subsequent deposited droplet behaviour on the surfaces revealed a dependence on wettability: on the hydrophilic carbon paper, captured droplets spread rapidly across the carbon paper, coalescing into a continuous liquid film that thickened over time. Once the film exceeded its gravitational stability, it detached and drained downward as continuous rivulets or dripping water, thereby sustaining a steady removal of collected fog. In contrast, on the hydrophobic carbon paper, incoming droplets adhered to discrete, nearly spherical drops with limited spreading. These droplets remained largely isolated, occasionally merging with neighbouring droplets, but rarely forming a stable film. As deposition continued, the droplets grew until reaching a critical size, at which point they detached from the surface. The detachment often occurred in a sliding motion, during which the moving droplet induced small-scale flooding, entraining other adhered droplets along its path and producing intermittent bursts of water removal. Taken together, hydrophilic surfaces promote continuous film formation and steady drainage, whereas hydrophobic surfaces maintain discrete droplets with episodic shedding events, leading to different modes of water collection. High-speed camera observations also reveal that droplet re-entrainment is completely suppressed under a strong electric field, such as at 12 kV. The high-speed camera video clips for all carbon papers are available in the electronic supplementary material.

### 3.4. Electrostatic fog collection efficiency

**Figure 8.**
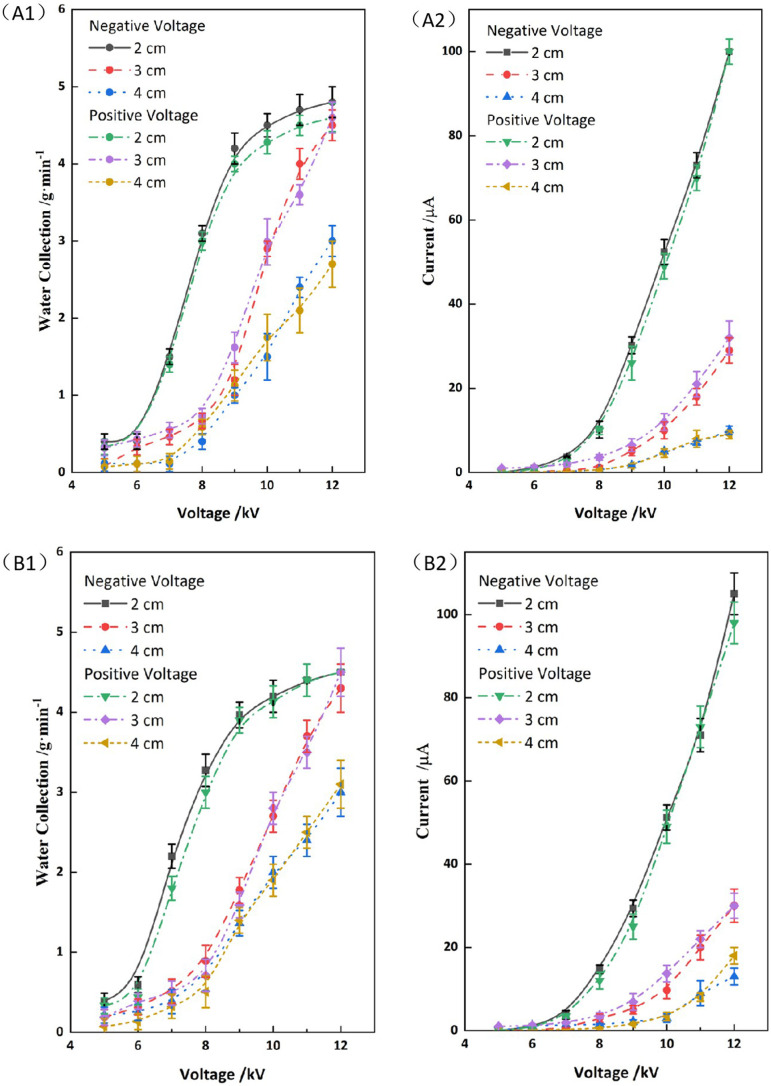
Correlations of fog collection efficiency and electrostatic current with applied voltages. The measurements were performed with collection distances of 2, 3 and 4 cm. (A) Hydrophilic carbon paper and (B) 40% PTFE-coated carbon paper. (A1,B1) Efficiency versus voltage (positive and negative). (A2,B2) Efficiency versus electrostatic current. Additional results for other PTFE-coated surfaces are provided in the electronic supplementary material.

The experimental results revealed that all carbon paper surfaces exhibited comparable collection efficiencies and currents. Therefore, hydrophilic carbon paper (figure 8A) and 40% PTFE carbon paper (figure 8B) were selected as examples, with the results for other carbon papers provided in the electronic supplementary material. Figure 8A,B shows that the hydrophilic and hydrophobic carbon surfaces exhibit similar collection efficiencies and electrostatic currents. Specifically, figure 8A1,B1 plots fog collection efficiency (in g min^−1^) versus voltage at different collection distances, while figure 8A2,B2 plots the correlation of electrostatic current with the applied voltage. In the absence of an electric field, fog particles exhibited random and sluggish movement, resulting in a collection efficiency of less than 0.1 g min^−1^. For different collection distances, significant electrostatic currents emerge only when the voltage exceeds a certain threshold, which is dependent on the collection distance.

At a collection distance of 2 cm, the applied voltages of ±5 and ±6 kV yielded electrostatic currents below 1 µA, corresponding to limited collection efficiencies of approximately 0.4 g min^−1^. When the voltage increased to ±7 kV, both current (3.0–4.0 µA) and collection efficiency (1.5–2.2 g min^−1^) exhibited a marked enhancement, indicating the onset of efficient electrostatic capture. A further increase to ±8 kV resulted in substantially higher currents (9.0–13.0 µA) and efficiencies (3.0–3.3 g min^−1^). At ±9 and ±10 kV, the current rose sharply to 23–35 and 45–58 µA, respectively, while the corresponding efficiencies reached 3.8–4.2 and 4.1–4.6 g min^−1^. Notably, despite the significant increase in current at ±10 kV compared to ±9 kV, the improvement in collection efficiency was marginal, suggesting the onset of a saturation regime. At higher voltages (±11 and ±12 kV), the collection efficiency plateaued at approximately 4.5 g min^−1^, further confirming the tailing-off effect due to charge saturation and droplet fission phenomena. As the collection distance increased, both the efficiency and the current decreased significantly. At an electrode distance of 3 cm, the collection efficiency decreases to below 1.0 g min^−1^ when the applied voltage is lower than 8 kV. In contrast, the efficiencies are approximately 1.3–1.8, 1.9–2.7, 3.4–3.8 and 4.1–4.5 g min^−1^ for applied voltages of ±9, ±10, ±11 and ±12 kV, respectively. Notably, at ±12 kV, the collection efficiency curve intersects with that of the 2 cm collection distance. At 4 cm, the efficiency was approximately 1, 2, 2.5 and 3.0 g min^−1^ for ±9, ±10, ±11 and ±12 kV, respectively. For the current response, figure 8A2,B2 illustrates that the current increases with applied voltage, albeit with different slopes. Beyond 8 kV, the currents rise nearly linearly with voltage. The curve corresponding to the 2 cm collection distance exhibits the steepest increase, whereas the curves for 3 and 4 cm show progressively lower slopes. Taken together with the results from other PTFE-modified carbon papers (electronic supplementary material, figure S3), these findings demonstrate that surface wettability exerts only a minimal influence on electrostatic fog collection efficiency, as both hydrophilic and hydrophobic carbon papers exhibit comparable electrostatic currents. In contrast, increasing the collection distance markedly reduces the current, which consequently diminishes the collection efficiency.

The statistical analysis of current measurements was performed using the Kruskal–Wallis test, which is a non-parametric method suitable for comparing medians across multiple groups without assuming a normal distribution [39]. This test was applied to evaluate the influence of four factors—voltage, polarity, distance and PTFE level—on the current response. Among the tested factors, voltage and distance have a highly significant influence on collection efficiency and current, while polarity and PTFE level do not. The analysis process is provided in the electronic supplementary material.

**Figure 9.**
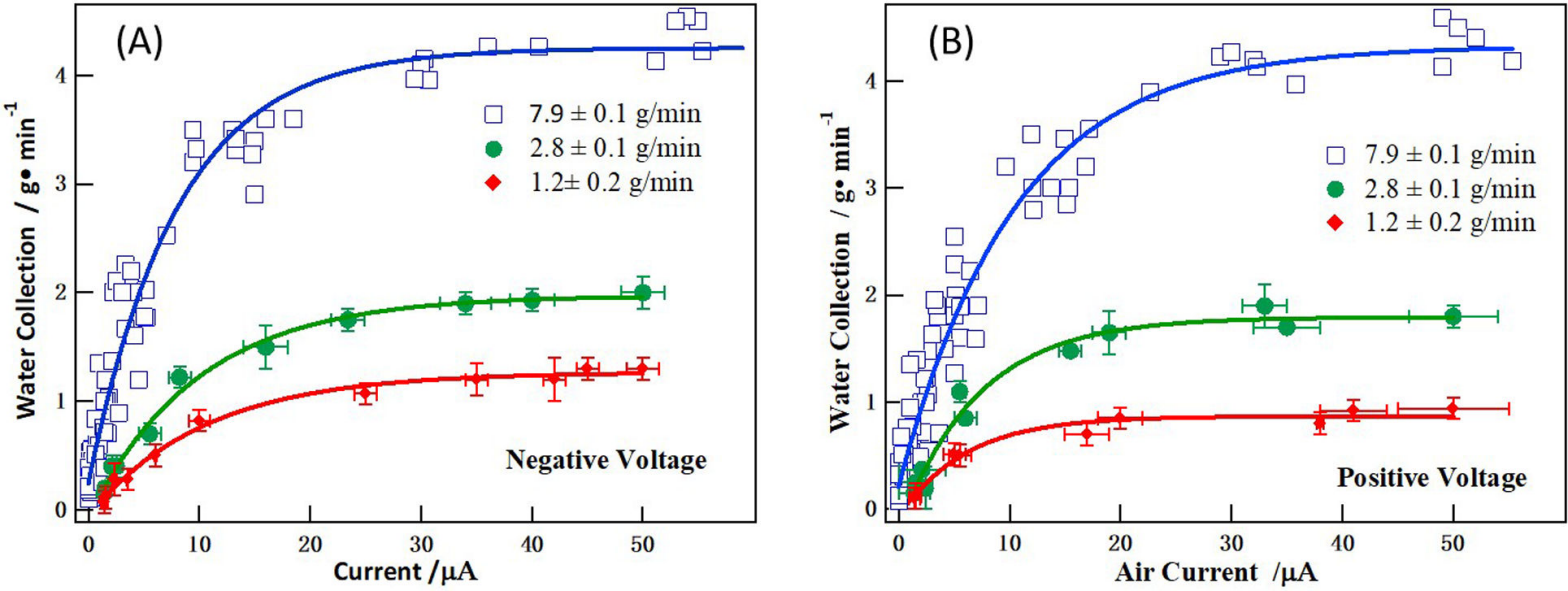
Correlations of fog collection efficiency with electrostatic current are shown for (A) negative voltage and (B) positive voltage. The fog stream flux speeds are 7.9 ± 0.1, 2.8 ± 0.1 and 1.2 ± 0.2 g min^−1^.

To elucidate the key factor governing the electrostatic fog collection efficiency, the correlations of fog collection efficiency with electrostatic currents are depicted in figure 9A for negative voltages and figure 9B for positive voltages. Three different fog streams of different flux speed are applied, which are 7.9 ± 0.1, 2.8 ± 0.1 and 1.2 ± 0.1 g min^−1^ to study flux speed influence. For a flux speed of 7.9 ± 0.1 g min^−1^, collection efficiency and the corresponding current values are adopted from figure 8A,B for three collection distances. The results reveal a clear correlation between the collection efficiency and electrostatic current. Initially, the collection efficiency increased sharply with increasing current, but the rate of increase diminished beyond 15–20 µA and gradually levelled off. This trend was consistent across the three different fog stream flux speeds, suggesting that the collection efficiency was primarily influenced by the current. For all three fog flux speeds, the collection efficiency could be fitted with a natural exponential function, and the fitting curves are shown in figure 9. In figure 9A, the experimental data under negative voltages were empirically fitted using natural exponential functions. The fitting equations are as follows: $y=4.3-4.0e^{-0.13}$ for the fog stream flux of 7.9 g min^−1^; $y=2.0-2.0e^{-0.10}$ for a flux of 2.8 g min^−1^ and $y=1.3-1.3e^{-0.095}$ for a flux of 1.2 g min^−1^. In figure 9B, the fitting equations are as follows: $y=4.3-4.1e^{-0.096}$ for a flux of 7.9 g min^−1^; $y=1.8-2.0e^{-0.14}$ for a flux of 2.8 g min^−1^ and $y=0.87-0.95e^{-0.17}$ for a flux of 1.2 g min^−1^.

The electrostatic fog collection plateau was also previously documented in wire-based electrostatic collectors [20] and multiple mesh collectors [40]. This tail-off phenomenon in collection efficiency can be attributed to charge saturation of fog droplets and subsequent Coulomb fission. In electrostatic fog collection, applying a high voltage initially promotes water capture by accelerating droplet migration; however, once the droplets approach the Rayleigh limit, they undergo Coulomb fission [41], fragmenting into smaller charged droplets that are more difficult to collect. The fragmentation leads to fog thinning and a plateau in collection efficiency despite further voltage increases. As shown in the high-speed camera snapshot in figure 7, at a high voltage of 12 kV, the fog stream becomes noticeably thinner, and the tail-off is obvious in figures 8 and 9 with the increasing applied voltage and currents.

To further demonstrate that the surface wettability of carbon paper plays a negligible role in electrostatic fog collection efficiency, the 0 wt% PTFE carbon paper was plasma-treated to reduce its water contact angle to approximately 15°, and its fog collection performance was subsequently investigated. The electrostatic fog collection efficiency of the plasma-treated carbon paper was evaluated and compared with that of a non-conductive PTFE film, as shown in electronic supplementary material, figure S4. At a collection distance of 2 cm, the fog collection of the plasma-treated carbon paper reached a plateau beyond 9 kV, exhibiting similar efficiency to that of other carbon paper samples, whereas the non-conductive PTFE film showed very limited fog deposition (<1 g min^−1^) across all applied voltages. These results serve as control experiments, highlighting that the electrical conductivity of the collection surface is essential for effective electrostatic fog deposition, while surface wettability has only a negligible influence on the collection efficiency.

In an electric field with sufficient ions, the fog particles absorb electrostatic ions. According to the analysis by Damark *et al.*, the saturation charge *q* of a fog particle can be calculated using equation (3.2) [6]:


(3.2) $$q=12\pi R_{d}^{2}\varepsilon_{0}\frac{V}{L},$$

where *R*_d_ is the diameter of the fog particles, *ε*_0_ is the permittivity of water, *V* is the high voltage of the electric field and *L* is the distance between the discharging electrode and the charge-receiving surface. At the collection surface, if a homogeneous electric field is assumed, the electric field strength *E* can be calculated using equation (3.3):


(3.3) $$E=\frac{V}{L}.$$

The electrostatic force acting on the charged fog particles is calculated as *F* = *Eq*, where *q* is the charge of the particles. This force facilitated the efficient capture of fog particles by overcoming air diffusion. In the absence of an electric field, fog particles exhibit random and slow movements. The electric field is the primary factor driving the deposition on the collection electrode surface. Therefore, the amount of collection *A* is linearly proportional to the work done by the electric field in moving the charged fog particles from the charge emitter to the carbon paper:


(3.4) $$A\propto W=FL=EqL=12\pi R_{d}^{2}\varepsilon_{0}\frac{V^{2}}{L}.$$

**Figure 10.**
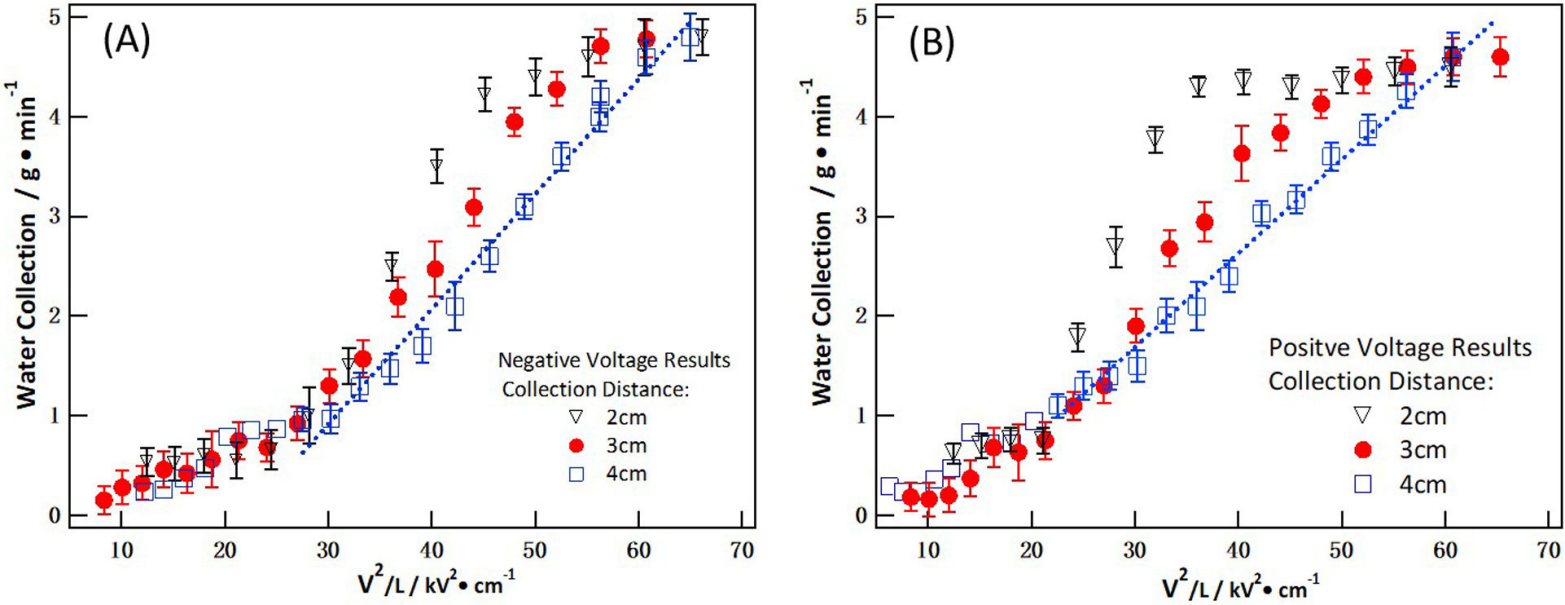
Correlation between collection efficiency and *V*²/*L* for pristine carbon paper: (A) for negative voltage and (B) for positive voltage. *V* represents the voltage and *L* is the fog collection distance between the charge emitters and the carbon paper collection surface.

Equation (3.4) indicates that the collection efficiency should exhibit a linear relationship with *V*^2^/*L*, assuming a homogeneous electric field, unsaturated charges on fog particle surfaces and negligible differences in collection time for the three collection distances. Figure 10 plots the collection efficiency versus *V*²/*L* for pristine carbon paper for negative (figure 10A) and positive (figure 10B) voltages. For both charges, at a collection distance of 4 cm, the water collection efficiency exhibited a more linear correlation with *V*²/*L*. However, at distances of 2 and 3 cm, the efficiency deviated from linearity. This deviation can be attributed to the stronger electric field at closer ranges, which exerts greater thrust on fog particles and the inhomogeneous electric field for shorter collection distances. The shorter the collection distance, the more pronounced the deviation, particularly at 2 cm. When *V*²/*L* reaches a certain value, charge saturation on the surface of the particles begins to affect the collection efficiency, resulting in a level-off, as shown in figure 10. According to equation (3.2), the electric field is more intense at shorter collection distances, and the inhomogeneity of the electric field can be more pronounced at shorter collection distances. This inhomogeneity could also account for the nonlinear trend observed in the collection efficiency versus *V*^2^/*L*.

**Figure 11.**
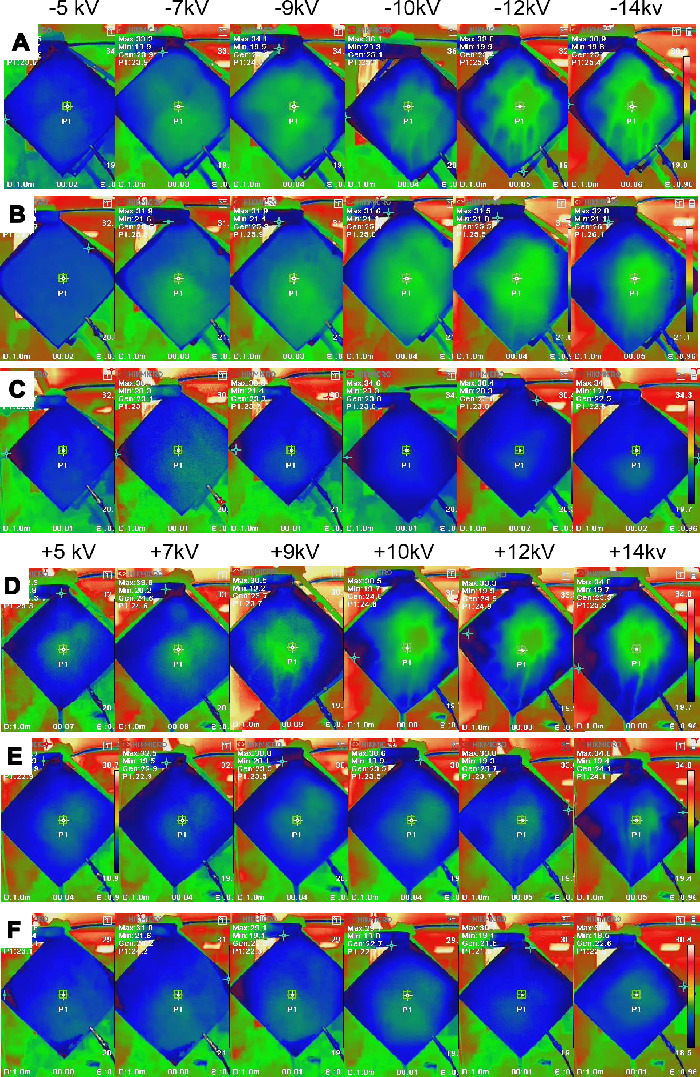
Thermal imaging analysis of temperature distribution on hydrophilic carbon paper during electrostatic fog collection at different voltages. (A–C) Negative voltages at collection distances of 2, 3 and 4 cm, respectively. (D–F) Positive voltages at the same collection distances. Columns from left to right correspond to applied voltages of ±5 kV, ±7 kV, ±9 kV, ±10 kV, ±12 kV, and ±14 kV.

To further examine the charged fog particle deposition zone on the carbon surface, a thermal camera was used to map the surface temperature distribution of the wetted carbon paper (figure 11). The colour scale is shown in the rightmost panel, where brighter hues denote higher temperatures. The region directly impacted by the fog stream exhibited a temperature comparable to that of the stream itself and appeared in bright green. In contrast, the surrounding background temperature was higher and displayed in red. Due to the porous structure of the carbon film, the adsorbed water was able to diffuse laterally into adjacent regions not directly exposed to the stream. Consequently, the transpiration cooling effect [42] causes these non-contact regions to display lower surface temperatures, as shown in blue, indicating a temperature below that of the stream (green). Therefore, the thermal camera can directly map the deposition zone of the fog stream on carbon paper. Figure 11 also illustrates how the trajectory of the charged fog stream changed as the electric field strength increased. For the 2 cm collecting distance (figure 11A,D), when the voltage was ±5, ±7 and ±9 kV, the charged fog stream impacted the surface uniformly with a vague cloud-shaped contact zone, and the contact zone area increased with voltage. As the voltage increases to ±10, ±12 and ±14 kV, the contact zone begins to concentrate and forms an irregular annular ring. In the central domain of the annular ring zone, the surface temperature was slightly higher than that of the fog stream and close to the environmental background temperature, suggesting that fog particles were distributed less in the centre of the fog stream under a strong electric field. Clearly, segregation of the charged fog stream from the current occurs under the strong influence of the electric field. At a collection distance of 3 cm (figure 11B,E), the contact zone appeared uniform for all applied voltages. The contact zone expanded with increasing voltage and reached its maximum at ±9 kV and ±10 kV. Beyond these voltages, the contact zone area began to contract as the voltage continued to rise. For a collection distance of 4 cm (figure 11C,F), smaller homogeneous deposition zones were observed at all applied voltages. Note that at ±12 and ±14 kV, water dripping was visible. In contrast, the absence of such traces under other conditions implies that collected water rapidly diffused to other areas of the porous carbon paper without forming dripping tracks. The surface temperature of the carbon paper was cross-verified using a K-type thermocouple, with results shown in electronic supplementary material, figure S5. Moist carbon paper exhibited an evaporative cooling effect of approximately 6 °C below the ambient-equilibrated water temperature. As a result, when a room-temperature fog stream contacted the carbon paper surface, the temperature difference between the deposition zone and non-contact zones could be readily visualized using the thermal camera.

Thermal camera images of the 40% PTFE sample under negative voltages are shown in electronic supplementary material, figure S6. At 2 cm, the charged fog stream is concentrated in a ring-like pattern, intensifying at higher voltages. At 3 and 4 cm, the deposition becomes more uniform, indicating a more homogeneous electric field. These patterns are similar to those observed for the 0% PTFE carbon paper.

## 4. Conclusions

This study investigated the influence of carbon paper surface wettability on electrostatic fog collection and evaluated the role of positive and negative electrostatic charges using a copper needle array as the charge dispenser. As the charge-receiving surface, the carbon papers demonstrated contact angles ranging from 15° to 150°, covering a broad spectrum of surface wettability. On hydrophilic surfaces, deposited fog droplets readily fused into a continuous water film that dripped down, whereas on PTFE-modified carbon papers, the collected fog formed discrete spherical droplets. Due to strong CAH, these droplets adhered to the hydrophobic surfaces until reaching a critical mass, at which point they rolled off. Despite these distinct deposition modes, the overall collection efficiency was largely unaffected. The PTFE-coated (non-wettable) carbon papers proved equally as effective for electrostatic fog collection as their hydrophilic counterparts. These results demonstrate that deposition efficiency is essentially independent of surface wettability, as both hydrophilic and PTFE-modified carbon papers exhibited comparable collection efficiencies under identical collection distances and applied voltages. Similarly, the electrostatic current measured on the carbon papers showed negligible dependence on surface wettability. The fog collection efficiency exhibited a strong dependence on collection distance, decreasing sharply as the distance increased, consistent with the distance-dependent attenuation of the electrostatic current. In contrast, the type of applied charge had little influence on deposition efficiency, as both negative and positive voltages yielded nearly identical collection efficiencies and electrostatic currents. These results highlight electrostatic current as the primary factor governing collection performance. Although efficiency initially rises with increasing current, it stabilizes once a critical threshold is reached, resulting in diminishing returns at higher currents. In addition, the inherent inhomogeneity of the electric field causes charged fog droplets to deviate from the main current trajectory, further influencing the spatial distribution of collection. The corresponding statistical analysis supports these conclusions.

The findings of this study have potential applications in several aspects. First, they can inform the design of more practical electrostatic fog collection devices; for instance, instead of conventional metal electrodes, lightweight carbon paper without additional hydrophilic or hydrophobic modification can serve as an effective charge-receiving surface electrode. Second, the proper design of the distance between the discharging electrode and the charge-receiving surface is crucial, as larger distances require higher voltages to sustain the electrostatic current, with the current being the dominant factor governing fog collection efficiency. Finally, identifying the voltage threshold is essential, as beyond this point, further increases in voltage yield only marginal improvements in collection efficiency. For future work, optimizing the electrode configuration to achieve a more homogeneous electric field distribution represents an important research direction.

## Data Availability

All relevant data are within the paper and its supplementary material [43].
